# Intracellular and Intercellular Gene Regulatory Network Inference From Time-Course Individual RNA-Seq

**DOI:** 10.3389/fbinf.2021.777299

**Published:** 2021-11-11

**Authors:** Makoto Kashima, Yuki Shida, Takashi Yamashiro, Hiromi Hirata, Hiroshi Kurosaka

**Affiliations:** ^1^ College of Science and Engineering, Aoyama Gakuin University, Sagamihara, Japan; ^2^ Department of Orthodontics and Dentofacial Orthopedics, Osaka University, Suita, Japan

**Keywords:** gene regulatory network, bulk RNA-seq, intracellular, time course, mouse

## Abstract

Gene regulatory network (GRN) inference is an effective approach to understand the molecular mechanisms underlying biological events. Generally, GRN inference mainly targets intracellular regulatory relationships such as transcription factors and their associated targets. In multicellular organisms, there are both intracellular and intercellular regulatory mechanisms. Thus, we hypothesize that GRNs inferred from time-course individual (whole embryo) RNA-Seq during development can reveal intercellular regulatory relationships (signaling pathways) underlying the development. Here, we conducted time-course bulk RNA-Seq of individual mouse embryos during early development, followed by pseudo-time analysis and GRN inference. The results demonstrated that GRN inference from RNA-Seq with pseudo-time can be applied for individual bulk RNA-Seq similar to scRNA-Seq. Validation using an experimental-source-based database showed that our approach could significantly infer GRN for all transcription factors in the database. Furthermore, the inferred ligand-related and receptor-related downstream genes were significantly overlapped. Thus, the inferred GRN based on whole organism could include intercellular regulatory relationships, which cannot be inferred from scRNA-Seq based only on gene expression data. Overall, inferring GRN from time-course bulk RNA-Seq is an effective approach to understand the regulatory relationships underlying biological events in multicellular organisms.

## Introduction

Regulation of gene expression is a fundamental factor that controls cellular events such as proliferation and differentiation. Understanding gene regulatory networks is important to elucidate the molecular mechanisms underlying cellular events. Recently, gene regulatory network (GRN) inference based on time-course data has garnered considerable attention in single-cell RNA-Seq (scRNA-Seq). State-of-the-art scRNA-Seq analysis techniques can generate transcriptome information from thousands of cells (Sasagawa et al., 2013; Klein et al., 2015; Hayashi et al., 2018; Sasagawa et al., 2018; Gao et al., 2020). Transcriptomic heterogeneity of cells due to asynchronous progression of cellular events enables us to infer regulatory relationships of genes. During inference, first, dimensional reduction of scRNA-Seq data provides a trajectory of cellular events such as differentiation and proliferation (Treutlein et al., 2014; Haghverdi et al., 2016). Then, assignment of pseudo-time can place cells along the trajectory. As scRNA-Seq with pseudo-time is a dense time-course observation of cellular events, gene regulatory networks can be inferred by comparing the timing of gene upregulation and downregulation along pseudo-time (Matsumoto et al., 2017; Aalto et al., 2020).

Originally, GRN inference was applied for gene expression data from tissue and pooled cells (bulk samples) generated using DNA microarray and RNA-Seq (Fernandez-Valverde et al., 2018; Ko and Brandizzi, 2020). Compared to steady-state data, time-course data enables GRN inference by comparing the timing of gene upregulation and downregulation (Krouk et al., 2010; Ogami et al., 2012; Iglesias-Martinez et al., 2016; Zhang et al., 2019). However, GRN inference from time-course data of bulk samples is not popular due to the following drawbacks: 1) RNA extraction and library preparation of a large number of bulk samples before sequencing are expensive and time-consuming (Yoshino et al., 2020), and 2) biological variances may result in inconsistencies between actual sampling time and transcriptome status (Kilfoil et al., 2009). Recent technical advances related to bulk RNA-Seq have overcome these limitations. Advances in sequencing platforms (Muir et al., 2016), RNA extraction method (Yoshino et al., 2020; Ujibe et al., 2021), and bulk 3′ RNA-Seq library preparation methods (Alpern et al., 2019; Kamitani et al., 2019; Li et al., 2020) have enabled a cost-effective time-course individual RNA-Seq (Kashima et al., 2020) (a time-series RNA-Seq targeting an entire embryo or tissue of each individual). Pseudo-time analysis for individual RNA-Seq might capture individual differences in the progression speed of biological events such as development. Following the assignment of pseudo-time to each individual RNA-Seq data, GRN can be inferred similar to an inference based on scRNA-Seq.

Theoretically, GRN inferred from whole-body and tissue RNA-Seq are different from those inferred from scRNA-Seq. scRNA-Seq provides transcriptomic information at the cellular level that enables inference of intracellular GRN involved in proliferation and differentiation (Lam et al., 2016). In contrast, bulk RNA-Seq of the entire body and tissues could contain transcriptomic information at the cell population level. Time course individual RNA-Seq during development would enable inference of both intracellular and intercellular GRN (cell–cell communications) involved in the developmental process. For instance, during embryonic development, the upregulation of ligand-related genes can be followed by the upregulation of downstream genes in cells expressing receptor genes (Basson, 2012).

Thus, we hypothesized that GRNs inferred from time-course individual RNA-Seq during embryonic development would include intercellular regulatory relationships between ligand genes and downstream genes of related signaling pathways. To test this hypothesis, we conducted time-course bulk RNA-Seq of individual mouse embryos during early development, followed by pseudo-time analysis and GRN inference.

## Materials and Methods

### Maintenance of Mice

Embryos were collected from pregnant female Institute of *Cancer* Research (ICR) mice (CLEA, Tokyo, Japan) at different stages (E7.5, E8.5, E9.5, E10.5, E11.5, E12.5, and E.13.5). The number of replicates (embryos) was 10 at E7.5 and 11 in the remaining stages. All sacrificed female mice were housed under a 12-h dark–light cycle, with the light phase starting from 8 am. All animal experiments were performed in accordance with the guidelines of the Animal Care and Use Committee of Osaka University Graduate School of Dentistry, Osaka, Japan. All experimental protocols were approved by Animal Care and Use Committee of Osaka University Graduate School of Dentistry. All methods are reported in accordance with the ARRIVE guidelines (Percie du Sert et al., 2020).

### RNA-Seq Extraction

The total RNA was extracted using the RNeasy^®^ kit (QIAGEN, Hilden, Germany) according to manufacturer’s protocol. The total RNA concentration was measured using the Qubit™ RNA HS Assay Kit (Thermo Fisher Scientific, Waltham, MA, United States) and was adjusted to 5 ng/μL and stored at −80°C until the subsequent analysis.

### RNA-Seq Library Preparation and Sequencing

Non targeted RNA-Seq was conducted according to the Lasy-Seq ver. 1.1 protocol (https://sites.google.com/view/lasy-seq/) (Kamitani et al., 2019; Kashima et al., 2020). Briefly, 50 ng of total RNA was reverse transcribed using an reverse transcription (RT) primer with index and SuperScript IV reverse transcriptase (Thermo Fisher Scientific). Thereafter, all RT mixtures were pooled and purified using an equal volume of AMpure XP beads (Beckman Coulter, Brea, CA, United States) according to the manufacturer’s instructions. Second strand synthesis was conducted with the pooled samples using RNaseH (5 U/μL; Enzymatics, Beverly, MA, United States) and DNA polymerase I (10 U/μL; Enzymatics). To avoid the carryover of large amounts of rRNAs, the mixture was subjected to RNase treatment using RNase T1 (Thermo Fisher Scientific). Subsequently, the samples were purified using 0.8× volume of AMpure XP beads. Fragmentation, end-repair, and A-tailing were conducted using 5× WGS Fragmentation Mix (Enzymatics, Beverly, MA, United States). The Adapter for Lasy-Seq was ligated using 5× Ligation Mix (Enzymatics, Beverly, MA, United States), and the adapter-ligated DNA was purified twice with 0.8× volume of AMpure XP beads. After optimizing the PCR cycles for library amplification by qPCR using EvaGreen, 20× in water (Biotium, Fremont, CA, United States) and the QuantStudio5 Real-Time PCR System (Applied Biosystems, Waltham, MA, United States), the library was amplified using KAPA HiFi HotStart ReadyMix (KAPA BIOSYSTEMS, Wilmington, MA, United States) on the ProFlex PCR System (Applied Biosystems, Waltham, MA, United States). The amplified library was purified with an equal volume of AMpure XP beads. One microliter of the library was subjected to electrophoresis using Bioanalyzer 2,100 with the Agilent High Sensitivity DNA kit (Agilent Technologies, Santa Clara, CA, United States) to assess quality. Subsequently, sequencing of 150-bp paired-end reads was performed using HiSeq X Ten (Illumina, San Diego, CA, United States).

### Mapping and Gene Quantification

Read 1 reads were processed with fastp (version 0.21.0) (Chen et al., 2018) using the following parameters: --trim_poly_x -w 20 --adapter_sequence = AGA​TCG​GAA​GAG​CAC​ACG​TCT​GAA​CTC​CAG​TCA --adapter_sequence_r2 = AGA​TCG​GAA​GAG​CGT​CGT​GTA​GGG​AAA​GAG​TGT -l 31. The trimmed reads were then mapped to the mouse reference sequences of Mus_musculus.GRCm38.cdna.all.fa, using BWA mem (version 0.7.17-r1188) (Li and Durbin, 2009) with the default parameters. The read count for each gene was calculated with salmon using -l IU, which specifies the library type (version v0.12.0) (Patro et al., 2017). Thereafter, using R (version 4.0.1) (ore Team. R (2015). A, 2015), the sum of read counts per gene was calculated. Genes with read counts greater than zero were used in the subsequent analysis.

### Pseudo-Time Analysis

**Read counts were normalized using the “NormalizeData” function with the default parameters in** Seurat (version 4.0.0) (Hao et al., 2020), which produces natural-log transformed (read per 10,000 + 1). For principal component analysis (PCA), the normalized read counts were centered but not scaled using the “ScaleData” function with the default parameters except for do.scale = F. PCA was then performed using the “RunPCA” function for genes with high dispersion, which were selected using the “FindVariableFeatures” function with default parameters except for selection.method = “mvp”. Finally, SingleCellExperiment (version 1.10.1) (Amezquita et al., 2020) and slingshot (version 1.6.1) (Street et al., 2018) were used to calculate the pseudo-time for each sample.

### Evaluation Considering Pseudo-Time Instead of Stage

The “smooth.spline” function in R (version 4.0.1) (ore Team. R (2015). A, 2015) with the default parameters except for “all.knots = T, lambda = 0.001” was used to obtain smoothed curve for normalized expression of each gene in the “data” slot of the Seurat object, and stage or pseudo-time. Then sum of squared residuals (SSR) between the observed and fitted values was calculated for each gene. The mean of SSRs was calculated against all genes and the high variable genes obtained with the “FindVariableFeatures” function.

### Gene Regulatory Network Inference

In the SCODE algorithm (Matsumoto et al., 2017), normalized expression data in the “data” slot of the Seurat object, and pseudo-time were used to infer GRN. *A* was optimized 20 times with 100 iterations and *D = 4*. Pearson’s correlation coefficients between values of each *A* from the 20 optimizations and the mean*A* (the average of each value of *A*) were calculated. In the following analysis, we used the average values of the top 10 *A* showing higher correlations with the mean*A* from 20 optimizations*.* To define the thresholds for downstream gene selection for each gene, the linear function was regressed using the “nls” function in R for the scatter plot of absolute values of *A* for downstream genes in the decreasing order; the *X* and *Y* axes represented the integers from 1 to 28,117, and the absolute values of *A*, respectively. Genes with larger absolute values of *A* than the Y values of the regressed line were defined as the inferred gene downstream of each gene.

In the dynGENIE3 algorithm (Huynh-Thu and Geurts, 2018), normalized expression data in the “data” slot of the Seurat object, and samples stage were used to infer GRN. E7.5, E8.5, E9.5, E10.5, E.11.5, E12.5, and E.13.5 were converted to 1, 2, 3, 4, 5, 6, and 7, respectively. All parameters were used with the default values. weight.matrix inferred by dynGENIE3 was used as inferred regulatory relationships.

### Evaluation of Inferred GRN by Comparing With a TF-Downstream Gene Database and Analyzing Downstream Genes of Ligand- and Receptor-Related Genes

To validate the inferred GRN, information regarding the binding motifs and targets for 438 mouse TFs in the TF2DNA database was used (Berger et al., 2006; Matys et al., 2006; Berger et al., 2008; Badis et al., 2009; Wei et al., 2010; Chen et al., 2011; Jolma et al., 2013; Sebé-Pedrós et al., 2013; Weirauch et al., 2013; Mathelier et al., 2014; Pujato et al., 2014; Weirauch et al., 2014). The Area Under the Curve (AUC) values for downstream prediction based on the absolute values of *A* were calculated using the “performance” function in ROCR (version 1.0–11) (Sing et al., 2005). Statistical analysis of enrichment of the validated target genes among the inferred genes was conducted using the “fisher.test” function in R. Statistical analysis of overlapping of the inferred downstream genes of ligand- and receptor-related genes was conducted using the “enrichment_test” function in Rvenn (version 1.1.0) (Akyol, 2019). For all statistical tests, Benjamini-Hochberg (BH) correction was performed using the “p.adjust” function. The upset plots were drawn using the “upset” function in UpSetR (version 1.4.0) (Conway et al., 2017).

### Evaluation of Intracellular Co-Expression of Upstream and Downstream Genes

Based on the batch-corrected scRNA-Seq data of approximately 60,000 cells of high quality (Han et al., 2018), the average expression of each gene of 98 cell types was calculated with the “AverageExpression” function in Seurat. Thereafter, Pearson’s correlation coefficient (PCC) of normalized expression levels of upstream and downstream genes was calculated with the “cor” function in R. The inferred upstream and downstream genes showing PCC more than 0.4 were defined as co-expressed genes.

## Results

### Pseudo-Time Analysis of the Time-Course Bulk RNA-Seq of Mouse Embryos

**FIGURE 1:**
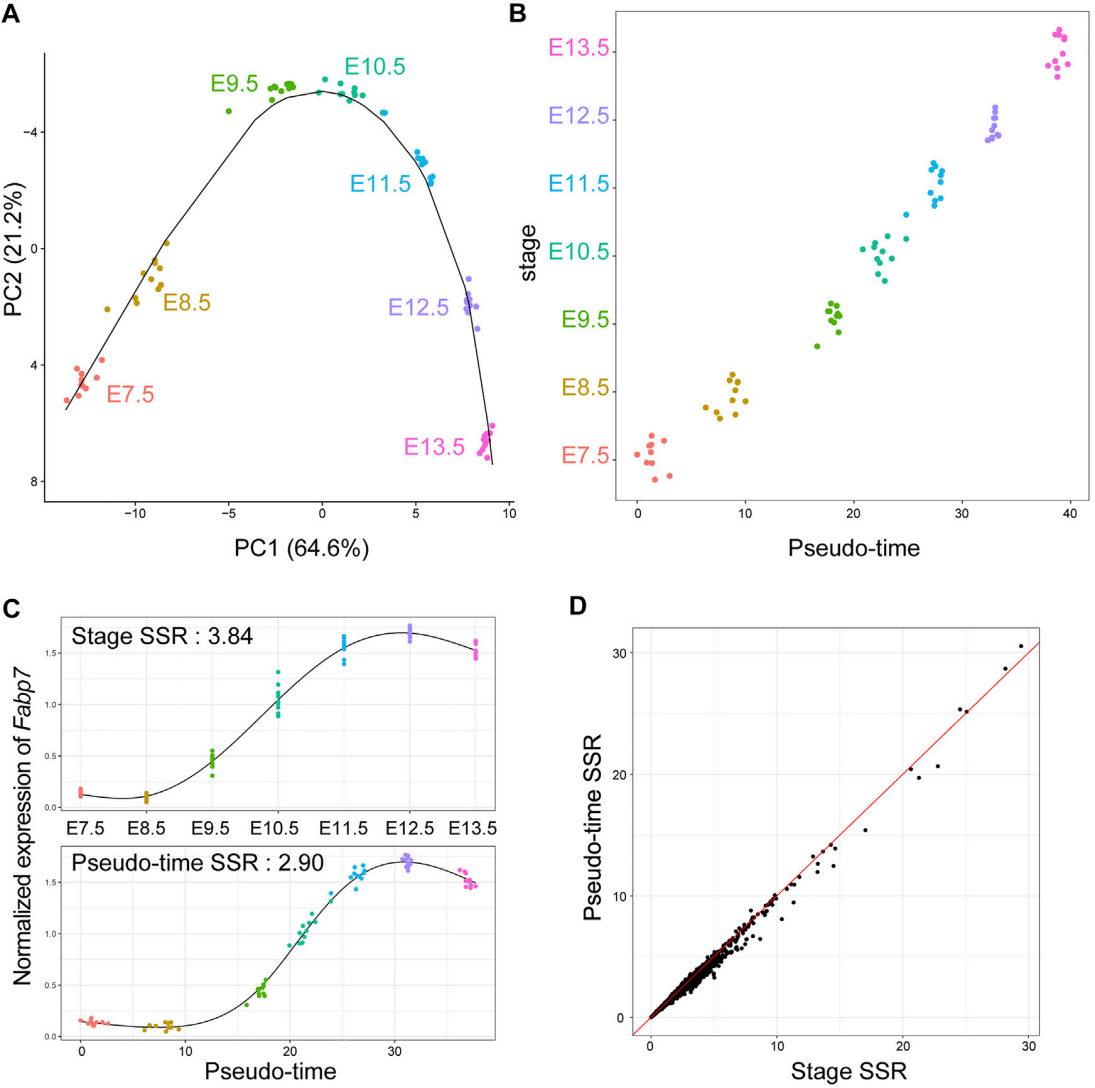
Pseudo-time analysis for time-course individual bulk RNA-Seq of mouse embryos in early development. **(A)** Transcriptomic trajectory of mouse embryos from E7.5 to E13.5. Each point on the PC1-PC2 plane indicates each individual RNA-Seq result. The solid line indicates an inferred trajectory by slingshot. **(B)** A scatterplot of pseudo-time and corresponding stage for each sample. Each point indicates each individual RNA-Seq result and is jittered along the *Y*-axis. **(C)** An example (Fabp7) of difference in gene expression dynamics along stage and pseudo-time. The black lines indicate smoothing curves for each data. Sum of squared residuals (SSRs) between the observed and fitted values were calculated. **(D)** A scatterplot of SSR of normalized gene expression of all genes along stage and pseudo-time. The red line indicates the same value between stage and pseudo-time.

To determine whether GRN inference based on time-course bulk RNA-Seq is effective, we conducted time-course bulk RNA-Seq for individual mouse embryos. RNA was extracted from each individual embryo (*n* = 10 or 11) at seven time points: E7.5, E8.5, E9.5, E10.5, E11.5, E12.5, and E.13.5, followed by 3′ RNA-Seq using the Lasy-Seq method (Kamitani et al., 2019). As a result, we obtained 76 RNA-Seq datasets with an average of 8.5 million reads per sample. The reads were mapped onto the mouse reference sequence, and then the read counts of each gene in each sample were calculated. We then used Seurat (Stuart et al., 2019), an R package for single cell omics analysis, for the normalization of read counts, detection of highly variable genes, and dimension reduction of omics date. On the PC1 and PC2 planes obtained with Seurat, samples in the same stage were close to each other (Figure 1A). As expected, clusters of each stage were ordered according to the developmental process (Figure 1B). Using the R package “slingshot” (Street et al., 2018), we inferred the developmental trajectory of mouse embryos and calculated the pseudo-time for each sample (Figures 1A,B). The pseudo-time analysis revealed individual differences among embryos in the speed of their developmental processes. For example, the pseudo-time of a sample at E10.5 and that at E11.5 were close to each other (Figures 1A,B). Using the pseudo-time analysis, the time-course RNA-Seq data of the seven time points could be converted into those of 76 time points. Using pseudo-time as temporal information instead of stage (real sampling time) improved the sum of squared residuals (SSRs) between the observed and fitted values (Figures 1C,D). The SSRs along the pseudo-time were decreased by 0.745% (all genes) and 3.835% (high variable genes), on an average, compared with the SSRs along stage. These results indicate that integrating pseudo-time into the analysis, instead of the actual sampled stage, could improve the capture of temporal expression dynamics, by considering individual differences in the progression speed of biological events during early embryonic development in mice.

### Gene Regulatory Network Inference Based on Individual RNA-Seq of Entire Mouse Embryos

**FIGURE 2:**
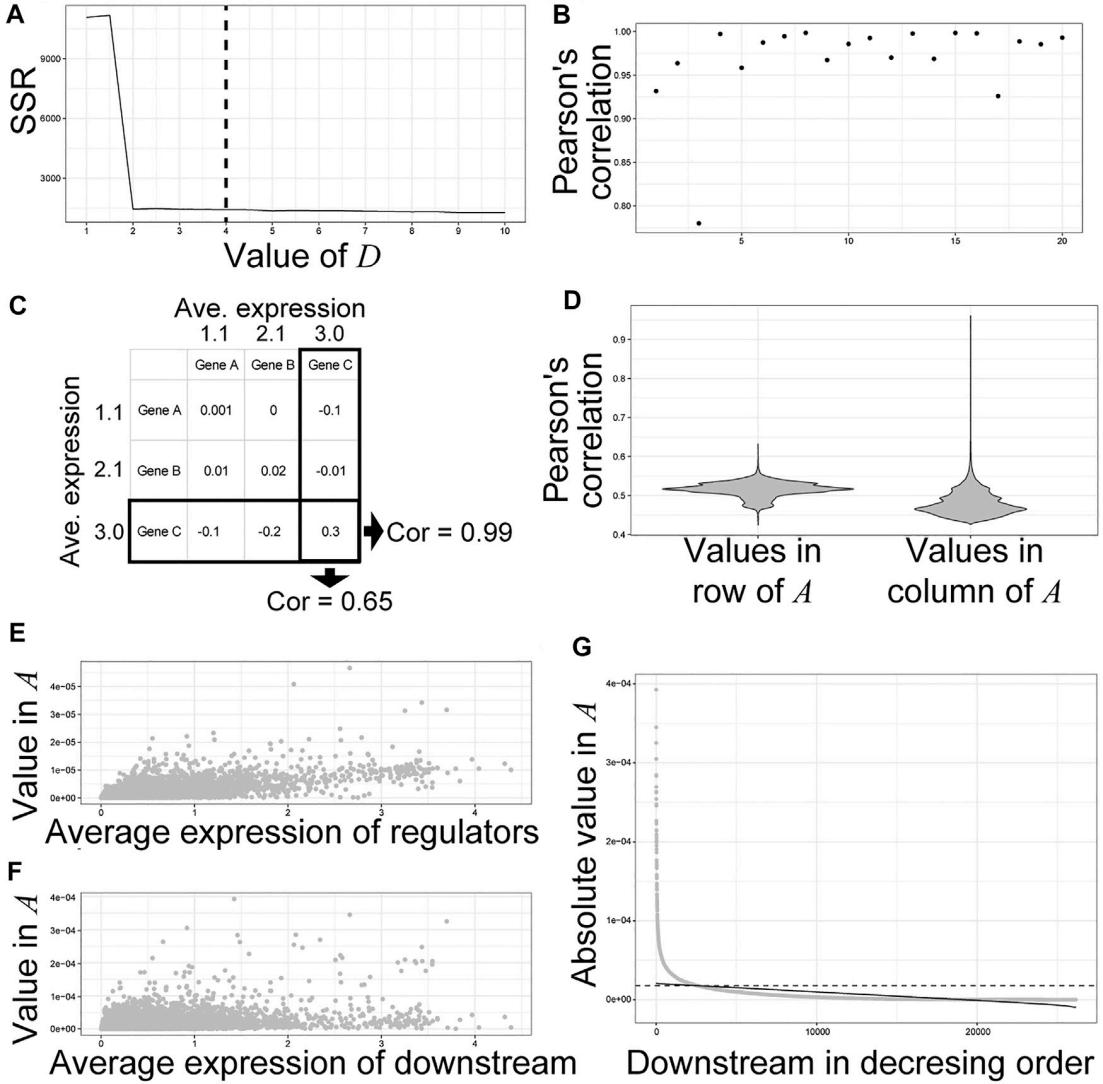
Inference of downstream genes based on the values in **(A)**. **(A)** Selection of a parameter D for SCODE. The sum of squared residuals (SSRs) for each D (from 1 to 10 by 0.5) was calculated. We used D = 4. **(B)** Pearson’s correlation coefficients between the values of each A from 20 optimizations and the meanA, which is the average of each value from all optimizations. **(C)** Calculation of Pearson’s correlations between the average expression level of each gene and the absolute values in each row and column of A. **(D)** The violin plots of Pearson’s correlations. **(E,F)** Scatter plot of the average expression level of each gene and the absolute values in the column **(E)** and row **(F)** of A for Sox8. **(G)** An example of the definition of thresholds for significant regulatory relationship between the regulator and downstream genes. A scatter plot of the values of A for a regulator, Sox8, in decreasing order. The solid line indicates a regression for the scatter plot. The dashed lines indicate the defined thresholds.

Next, we inferred a GRN from the dataset of time-course individual RNA-Seq of entire mouse embryos. We used SCODE, which solves linear ordinary differential equations to infer GRN (Matsumoto et al., 2017). To avoid incorrect emphasis on the technical noise of RNA-Seq, we used a non-scaled normalized gene expression matrix as the input for SCODE. Selection of the *D* size, a parameter that affects the number of assumed basic patterns of expression dynamics of the dataset, is important for robust GRN inference, as an unnecessarily large *D* causes an unstable inferred GRN (Matsumoto et al., 2017). In this study, we used *D =* 4 similar to that in a previous study (Matsumoto et al., 2017), with which the SSR was relatively small (Figure 2A). For 28,117 genes whose read counts were greater than zero, SCODE produced *A* (28,117 × 28,117 matrix) corresponding to the inferred gene regulatory network, in which the value of *A*_*i,j*_ indicates regulatory effects on the downstream gene *i* from the regulator *j*. *A*_*i,j*_ > 0 indicates that the regulator *j* positively regulates gene *i,* whereas *A*_*i,j*_ < 0 indicates the opposite. Because SCODE optimizes *A* by random sampling, we optimized *A* 20 times to check for reproducibility. Thereafter, PCCs between the values of each *A* from the 20 optimizations and the mean*A* were calculated (Figure 2B). Almost all optimizations produced a similar *A* with high correlation coefficients (Figure 2B)*.* In the subsequent analysis, we used the average values of the top 10 *A* showing higher correlations with the mean*A* from 20 optimizations*.* We then tried to define the thresholds for significant regulatory relationships between regulators and downstream genes. Because the average expression level of the downstream genes showed higher correlation with the absolute values of *A* compared to the regulators (Figures 2C,D), we independently defined the threshold for each regulator instead of a constant threshold previously used (Matsumoto et al., 2017). For example, the absolute values of *A* indicating regulatory relationships between *Sox8* and its regulators showed a positive correlation (PCC = 0.76) (Figure 2E), whereas the absolute values of *A* showing regulatory relationships between *Sox8* and its downstream genes showed smaller correlations (PCC = 0.54) (Figure 2F). Although most of the inferred values of *A* for regulatory relationships between *Sox8* and its downstream genes were around zero, some values were outliers (Figure 2G). To define the threshold for the downstream genes of *Sox8*, we regressed the linear function for the scatter plot of absolute *A* values for the downstream genes in the decreasing order and defined the threshold (Figure 2G). Finally, in the subsequent analysis, genes with larger absolute values of *A* than threshold were defined as the inferred genes downstream of *Sox8* (Figure 2G).

### Validation of the Inferred Network

**FIGURE 3:**
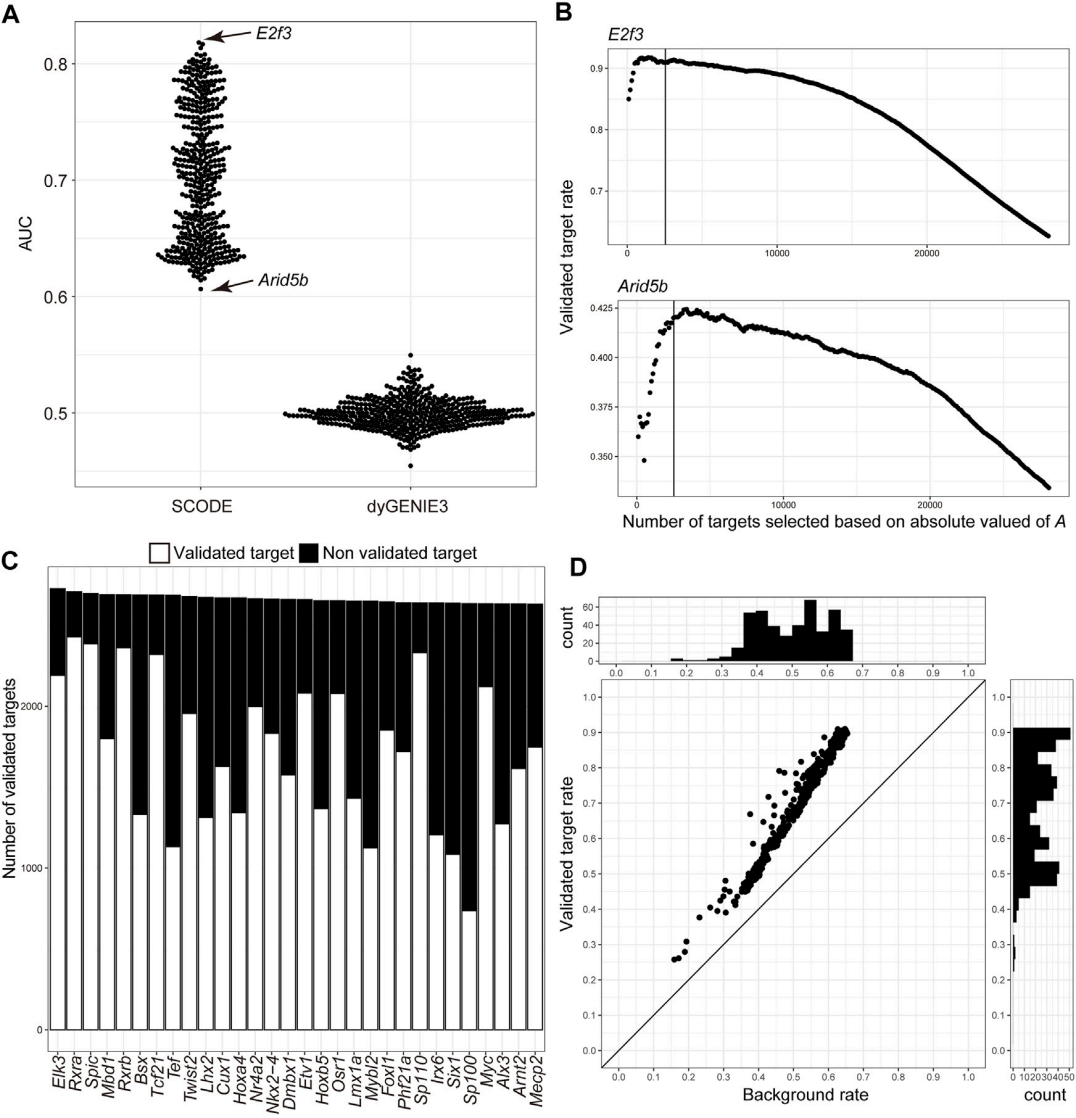
Validation of the inferred regulatory network of transcription factors with the TF2DNA database. **(A)** Scatter plot of area under the curve (AUC) target selection for each transcription factor (TF) based on the absolute values of A inferred using SCODE and weight.matrix inferred using dyGENIE3. **(B)** Scatter plots of the validated inferred target gene rate of E2f3 and Arid5b in the TF2DNA database. Downstream genes of TFs were selected based on the absolute values of A in decreasing order. Solid lines indicate genes at the thresholds. **(C)** Bar graph of validated and non-validated downstream genes of each TF in the TF2DNA database, in decreasing order of the total number of inferred downstream genes. Only the top 30 TFs are shown. **(D)** Scatter plot of the validated target gene rate of the inferred downstream genes of TFj and the background rate of target genes in the TF2DNA database. Histograms show the distribution of validated target gene rates of inferred downstream genes and the background rates of downstream genes in the TF2DNA database.

Next, we evaluated the inferred GRN by comparing the inferred genes downstream of the TFs with the information in the TF2DNA database, which is an experimental-source-based database of the binding motifs and downstream genes for 438 mouse TFs (Pujato et al., 2014; Badis et al., 2009; Wei et al., 2010; Weirauch et al., 2013; Berger et al., 2006; Mathelier et al., 2014; Matys et al., 2006; Weirauch et al., 2014; Berger et al., 2008; Chen et al., 2011; Jolma et al., 2013; Sebé-Pedrós et al., 2013). First, we evaluated the effectiveness of target prediction based on the absolute values of *A* by calculating the AUC (Figure 3A), and obtained an average AUC of 0.704, suggesting that the inferred regulatory relationships could reflect the actual regulatory network. Furthermore, we calculated the AUC for inferred regulatory relationships using dynGENIE3 (Huynh-Thu and Geurts, 2018), an algorithm that uses sampled stage information as temporal information to infer GRN. The average AUC was 0.50 (Figure 3A), suggesting the adequacy of GRN inference based on time-course individual RNA-Seq. Second, we examined the validity of the defined thresholds by assessing the differences between the validated target gene rate (number of validated target genes/number of all inferred downstream genes) above the defined thresholds and the best validated rate (Figure 3B and Supplementary Table S1). Below the threshold, the validated target gene rates for the 438 TFs were 0.69% smaller than the best validated target gene rates on an average. The validated target genes were 67.5% of the inferred downstream genes on an average (Supplementary Table S1, Figures 3C,D). Compared with the background rate (number of known target genes/number of all genes), the validated target gene rates of the inferred downstream genes of all TFs were statistically high (adjusted *p*-value < 0.01) (Figure 3D). These results suggest that our approach could infer the GRN underlying early development in mice.

### Inferred Network Contained Regulatory Relationships Involved in Cell–Cell Interaction

**FIGURE 4:**
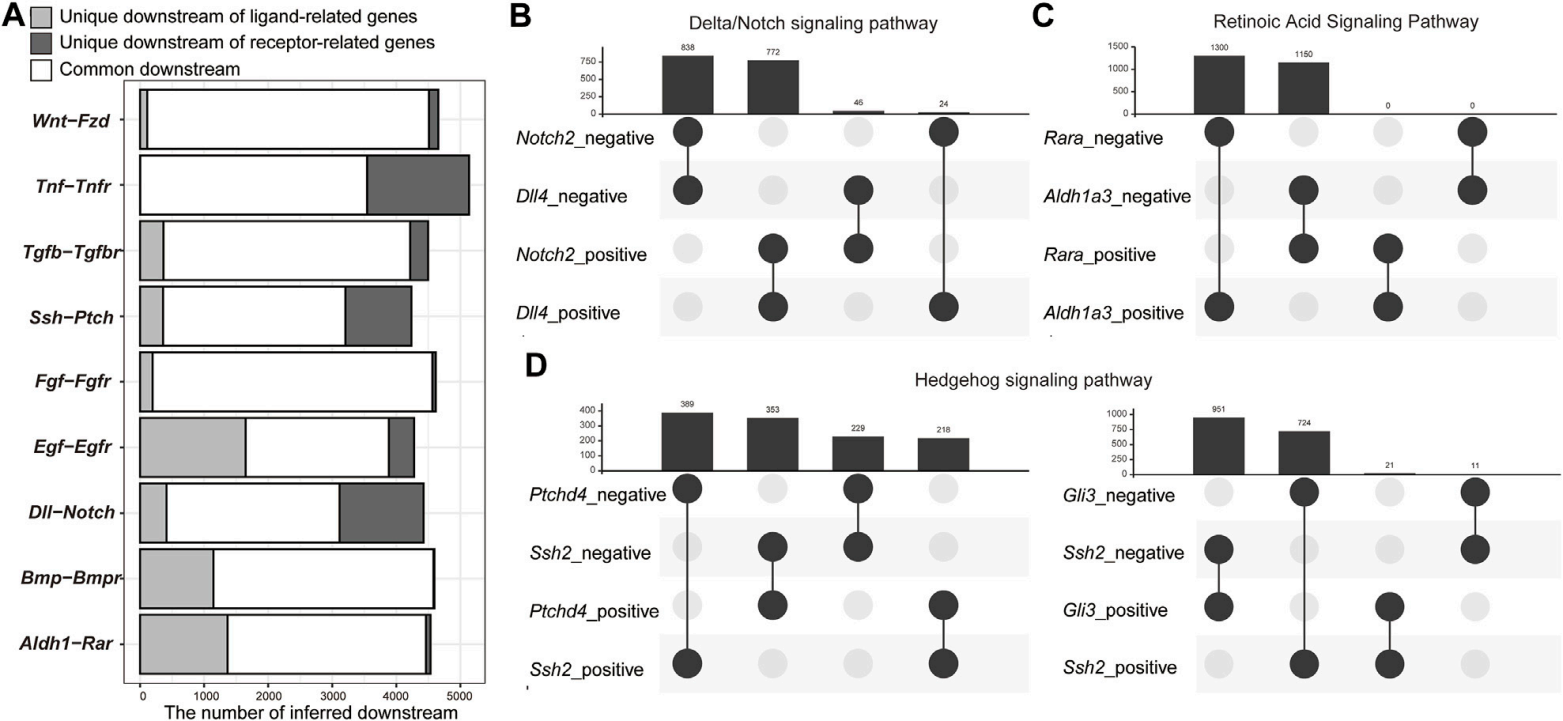
Overlap of inferred genes downstream of ligand- and receptor-related genes. **(A)** Bar plot of the number of inferred downstream genes that are common and unique for each ligand-receptor pair. **(B–D)** Upset plots of inferred downstream genes that are positively and negatively regulated by the representative ligand- and receptor-related genes. **(B)** Retinoic acid signaling pathway. **(C)** Delta/Notch signaling pathway. **(D)** Hedgehog signaling pathway.

Our GRN inference was based on bulk RNA-Seq containing the information of all cells in the body. We thus hypothesized that the inferred GRN also included the intercellular regulatory network. To examine this possibility, we checked the overlaps of inferred downstream genes of genes related to the ligands and receptors of nine major signaling pathways (Figure 4A): Wnt/βcatenin, TNF, TGF-β, Hedgehog, FGF, EGF, Delta/Notch, BMP, and retinoic acid (RA) signaling pathways (Supplementary Table S2). As expected, the inferred downstream genes of all pairs of ligand and receptor genes were significantly overlapped (adjusted *p*-value < 0.01) (Figure 4A). On an average, 94.2% of the inferred downstream genes of the ligand- and receptor-related genes were overlapped (Figure 4A). For example, 4,510 genes were inferred as downstream of *Wnt* genes and 4,545 genes were downstream of *Fzd* genes. However, 97.5% of the inferred downstream of *Wnt* genes were also inferred as the downstream of *Fzd* genes (Figure 4A). As SCODE can infer whether each downstream gene is positively or negatively regulated (Matsumoto et al., 2017), we assessed the overlaps of positively and negatively regulated gene downstream of the representative ligand-related and receptor-related genes of each signaling pathway, that is, with the highest number of inferred downstream genes among each gene family (Figures 4B–D, and Supplementary Figures S1, S2). For six signaling pathways (TNFβ, TGF, FGF, EGF, Delta/Notch, and BMP signaling pathways), which activate the downstream genes only when ligands bind to the receptors (Gilbert and Barresi, 2017), the regulatory directions of ligand- and receptor-related genes for most inferred downstream genes were the same (Figure 4B and Supplementary Figure S1). In contrast, in case of the RA signaling pathway, wherein the RA receptors function as transcriptional repressors without RA binding (Supplementary Figure S2A) (Glass and Rosenfeld, 2000), the regulatory directions of a ligand-related gene, *Aldh1a3,* which encodes a protein involved in RA synthesis, and a receptor-related gene, *Rara,* for the downstream genes were opposite (Figure 4C). In case of the Hedgehog signaling pathway, the regulatory directions of *Ssh2* and *Ptchd4* tended to be opposite (Figure 4D). The regulatory directions of *Ssh2* and *Gli3* for the downstream genes were also opposite (Figure 4D). In the absence of Hedgehog ligands, the full-length Gli family proteins are ubiquitinated and function as repressors (Supplementary Figure S2B) (Skoda et al., 2018). With the binding of Hedgehog ligands, PATCHED inhibits the degradation of the Gli family proteins and allows them to function as transcriptional activators (Supplementary Figure S2A) (Skoda et al., 2018). Thus, the overlap of downstream genes that are positively and negatively regulated by *Ssh2, Ptchd4,* and *Gli3* is reasonable. In case of the Wnt/βcatenin signaling pathway, the regulatory directions of *Wnt7a* and *Fzd5* for the downstream were opposite (Supplementary Figure S3A). Our time-course RNA-Seq revealed that among the *Fzd* and *Wnt* genes, *Fzd5* and *Wnt8a,* but not *Wnt7a,* were the only genes expressed dominantly in the early developmental stage among the protein families (Supplementary Figures S4, S5). Similar regulatory directions were found for most of the inferred downstream genes of *Wnt8a-Fzd5*, which could be a functional pair in the early developmental stages. Furthermore, the regulatory directions for most of the inferred downstream genes of *Wnt7a-Fzd6* (*Fzd* genes with the second most inferred downstream genes) were also the same (Supplementary Figure S3).

Next, we evaluated the co-expression of inferred downstream and upstream genes. Based on a publicly available mouse cell atlas (Han et al., 2018), PCCs of normalized expression levels of upstream and downstream genes were calculated (Supplementary Figure S6A), and the pairs of PPC >0.4 was defined as co-expressed genes. Although the ratios of co-expression were overall low due to limited characteristic of scRNA-Seq, the ratio of co-expression of ligand-related genes and their inferred downstream was significantly lower than that of receptor-related genes and TFs (Supplementary Figure S6B). This suggests that the inferred downstream genes of ligand-related genes tended to be expressed in cells that do not express ligand-related genes.

### Validation of Time-Course Individual RNA-Seq-Based Gene Regulatory Network Inference With a Publicly Available RNA-Seq Data

**FIGURE 5:**
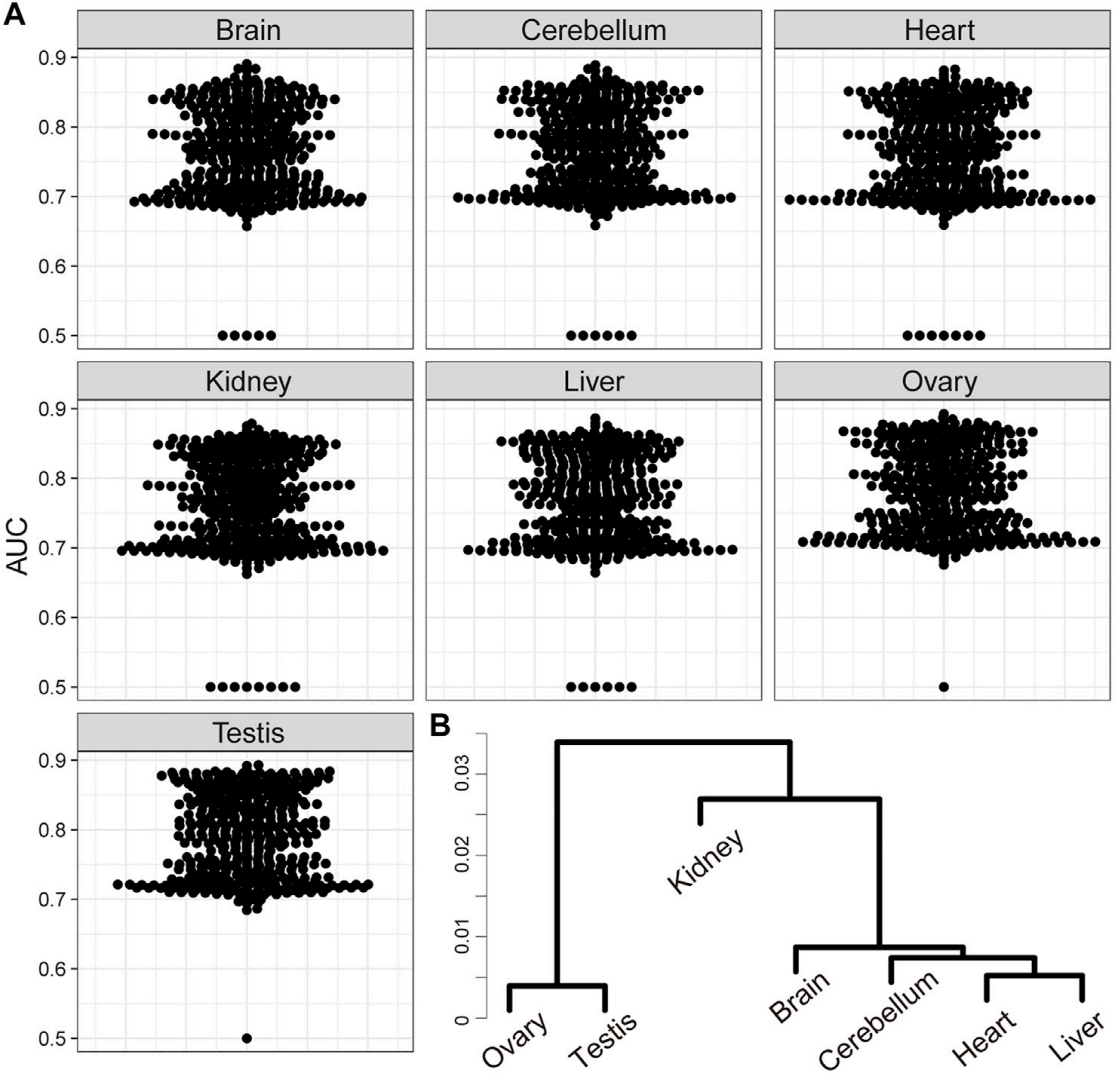
Validation of time-course individual RNA-Seq-based GRN inference with a public organ-level RNA-Seq data. **(A)** Scatter plots of area under the curve (AUC) target selection for each transcription factor (TF) based on the absolute values of A inferred from public time-course organ-level RNA-Seq data using SCODE. **(B)** Hierarchical clustering of the inferred GRNs from each organ-level RNA-Seq data.

Finally, we also inferred GRNs from a publicly available dataset of time-course organ-level individual RNA-Seq of mouse (Cardoso-Moreira et al., 2019). This dataset contained data of five somatic tissues (brain, cerebellum, hear, kidney, and liver) and two germline tissues (ovary and testis). The inferred relationships between TFs and downstream genes from each organ data tended to show higher AUC values for the TF2DNA database than those based on our time-course individual whole-embryo RNA-Seq (Figures 3A, 5A). In addition, same as the inferred GRN based on our whole-embryo RNA-Seq data, the inferred downstream genes of all pairs of ligand- and receptor-related genes were significantly overlapped (adjusted *p* < 0.01) (Supplementary Figure S7). The AUC values for several TFs from organ-level RNA-Seq were considerably worse than those from our RNA-Seq data (Figure 5A), suggesting organ-specificity of GRNs. As expected, hierarchical clustering revealed differences of the inferred GRNs between somatic and germline organs (Figure 5B).

In conclusion, our approach would allow successful inference of the intercellular regulatory relationships related to the major signaling pathways as well as the intracellular pathways related to TFs.

## Discussion

Recently, GRN inference based on the combination of scRNA-Seq and pseudo-time analysis has garnered considerable attention (Dai et al., 2020). However, to our knowledge, this study is the first to report GRN inference based on the combination of individual bulk RNA-Seq and pseudo-time analysis. Here, pseudo-time explained variances of gene expression during early development in mice better than the actual sampled stage (Figures 1C,D), suggesting that the variance of gene expression observed using our time-course individual RNA-Seq was not mere stochastic variability in gene expression but individual difference in the progression speed of development. Thus, pseudo-time uses the correlation of gene expression dynamics more effectively than the actual temporal information in time-course individual RNA-Seq.

Unlike scRNA-Seq, which can elucidate transcriptomic dynamics in a certain cellular event such as proliferation and differentiation, bulk RNA-Seq could provide a mixture of various transcriptomic dynamics regarding the cellular events occurring in an embryo (Chasman and Roy, 2017). Theoretically, GRN inference based on bulk RNA-Seq cannot provide multiple lineage-specific regulatory relationships. This may explain why GRN inference based on bulk RNA-Seq has not been attempted as with scRNA-Seq. In this study, we succeeded in inferring the known regulatory relationships of the TFs in the TF2DNA database with a high AUC compared with the GRN inferred from scRNA-Seq (Chen and Mar, 2018) (Figure 3). This suggests that GRN inference from bulk RNA-Seq provides insights into the regulatory interconnections of genes, even though GRN inferred from bulk RNA-Seq would have limitations in the comprehensiveness of inferred regulatory relationships compared with those from scRNA-Seq. Furthermore, substantial changes in the cell population occur during early development stages. It is possible that our GRN inferred from bulk RNA-Seq merely reflects the changes in cell populations but not the interconnections of genes. Considering the results of inference for the *Wnt* and *Fzd* genes (Supplementary Figure S2), these points should be considered when interpreting the inferred GRN based on the time-course bulk RNA-Seq.

Assignment of pseudo-time is an important step in GRN inference from time-course data. In case of scRNA-Seq, the accuracy of pseudo-time assignment is controversial (Tritschler et al., 2019). In contrast, in the case of time-course individual bulk RNA-Seq, the accuracy of pseudo-time assignment could be assured by the actual sampling time, which can be an advantage compared with GRN inference from scRNA-Seq.

Herein, we proposed a new strategy for the threshold of significant gene regulatory relationships inferred by SCODE (Figure 2). In the case of *E2f3,* the number of predicted target genes is only ∼10% of the actual targets with our strategy. Shifting the threshold to approximately 17,000 targets for *E2f3* may still result in a validated target rate of approximately 80% (Figure 3B). As decreasing the threshold may produce false positive inferred relationships for some genes, the determination of the threshold remains a challenge. Considering high AUCs for the TFs in the database (Figure 3A), the threshold should change depending on the aim of the study.

GRN indicates the intracellular interconnections of genes in a narrow sense; intercellular regulation of genes via cell–cell communication is also a key factor to understand the regulatory mechanisms underlying multicellular organisms. Several studies have attempted to systematically identify cell–cell communications based on single cell gene expression profiles and information regarding ligand–receptor pairs (Kumar et al., 2018; Wang et al., 2019; Cabello-Aguilar et al., 2021; Jin et al., 2021). As these approaches require prior knowledge, they could only be applied for the major model organisms and cannot reveal novel signaling pathways. We demonstrated that GRN inference based on time-course individual RNA-Seq could infer intercellular regulatory relationships related to cell–cell communication via cell signaling pathways. This approach only requires time-course RNA-Seq results and is applicable for non-model organisms without ligand–receptor information. Theoretically, the GRN inferred based on scRNA-Seq cannot include intercellular interconnections of genes. Intracellular co-expression knowledge of upstream and downstream genes, which could be obtained form scRNA-Seq experiments, would be useful for systematic identification of genes involved in cell–cell communications during cellular events of interest. Taken together, our approach is a powerful tool to understand intracellular and intercellular regulatory relationships of genes, which cannot be achieved using the existing GRN inferences based on scRNA-Seq alone. As discussed above, bulk RNA-Seq is limited in the comprehensiveness of inferred regulatory relationship as multiple lineage-specific regulatory relationships cannot be deduced from GRN inference based on bulk RNA-Seq. Higher AUC values of the inferred GRNs from organ-level RNA-Seq than whole-embryo RNA-Seq would reflect this limitation (Figures 3A, 5A). A future novel bioinformatic approach that can deconvolute gene expression in each tissue and cell lineage from bulk RNA-Seq will help overcome this limitation.

## Data Availability

The datasets presented in this study can be found in online repositories. The names of the repository/repositories and accession number(s) can be found below: https://www.ncbi.nlm.nih.gov/, PRJNA725414.

## References

[B1] Aalto A. Viitasaari L. Ilmonen P. Mombaerts L. Gonçalves J. (2020). Gene Regulatory Network Inference from Sparsely Sampled Noisy Data. Nat. Commun. 11, 3493. 10.1038/s41467-020-17217-1 32661225 PMC7359369

[B2] Akyol T. Y. (2019). RVenn: An R Package for Set Operations on Multiple Sets. Available at: https://cran.r-project.org/web/packages/RVenn/vignettes/vignette.html .

[B3] Alpern D. Gardeux V. Russeil J. Mangeat B. Meireles-Filho A. C. A. Breysse R. (2019). BRB-Seq: Ultra-Affordable High-Throughput Transcriptomics Enabled by Bulk RNA Barcoding and Sequencing. Genome Biol. 20, 71. 10.1186/s13059-019-1671-x 30999927 PMC6474054

[B4] Amezquita R. A. Lun A. T. L. Becht E. Carey V. J. Carpp L. N. Geistlinger L. (2020). Orchestrating Single-Cell Analysis with Bioconductor. Nat. Methods 17, 137–145. 10.1038/s41592-019-0654-x 31792435 PMC7358058

[B5] Badis G. Berger M. F. Philippakis A. A. Talukder S. Gehrke A. R. Jaeger S. A. (2009). Diversity and Complexity in DNA Recognition by Transcription Factors. Science 324, 1720–1723. 10.1126/science.1162327 19443739 PMC2905877

[B6] Basson M. A. (2012). Signaling in Cell Differentiation and Morphogenesis. Cold Spring Harb Perspect. Biol. 4, a008151. 10.1101/cshperspect.a008151 22570373 PMC3367549

[B7] Berger M. F. Badis G. Gehrke A. R. Talukder S. Philippakis A. A. Peña-Castillo L. (2008). Variation in Homeodomain DNA Binding Revealed by High-Resolution Analysis of Sequence Preferences. Cell 133, 1266–1276. 10.1016/j.cell.2008.05.024 18585359 PMC2531161

[B8] Berger M. F. Philippakis A. A. Qureshi A. M. He F. S. Estep P. W. Bulyk M. L. (2006). Compact, Universal DNA Microarrays to Comprehensively Determine Transcription-Factor Binding Site Specificities. Nat. Biotechnol. 24, 1429–1435. 10.1038/nbt1246 16998473 PMC4419707

[B9] Cabello-Aguilar S. Alame M. Kon-Sun-Tack F. Fau C. Lacroix M. Colinge J. (2021). SingleCellSignalR: Inference of Intercellular Networks from Single-Cell Transcriptomics. Nucleic Acids Res. 48, e55. 10.1093/nar/gkaa183 PMC7261168 32196115

[B10] Cardoso-Moreira M. Halbert J. Valloton D. Velten B. Chen C. Shao Y. (2019). Gene Expression Across Mammalian Organ Development. Nature 571, 505–509. 10.1038/s41586-019-1338-5 31243369 PMC6658352

[B11] Chasman D. Roy S. (2017). Inference of Cell Type Specific Regulatory Networks on Mammalian Lineages./pmc/articles/PMC5656272/ 10.1016/j.coisb.2017.04.001 PMC5656272 29082337

[B12] Chen L. Wu G. Ji H. (2011). hmChIP: A Database and Web Server for Exploring Publicly Available Human and Mouse ChIP-Seq and ChIP-Chip Data. Bioinformatics 27, 1447–1448. 10.1093/bioinformatics/btr156 21450710 PMC3087956

[B13] Chen S. Mar J. C. (2018). Evaluating Methods of Inferring Gene Regulatory Networks Highlights Their Lack of Performance for Single Cell Gene Expression Data. BMC Bioinformatics 19, 232. 10.1186/s12859-018-2217-z 29914350 PMC6006753

[B14] Chen S. Zhou Y. Chen Y. Gu J. (2018). Fastp: An Ultra-fast All-In-One FASTQ Preprocessor. Bioinformatics 34, i884–i890. 10.1093/bioinformatics/bty560 30423086 PMC6129281

[B15] Conway J. R. Lex A. Gehlenborg N. (2017). UpSetR: An R Package for the Visualization of Intersecting Sets and Their Properties. Bioinformatics 33, 2938–2940. 10.1093/bioinformatics/btx364 28645171 PMC5870712

[B16] Dai H. Jin Q. Q. Li L. Chen L. N. (2020). Reconstructing Gene Regulatory Networks in Single-Cell Transcriptomic Data Analysis. Zool Res. 41, 599–604. 10.24272/j.issn.2095-8137.2020.215 33124218 PMC7671911

[B17] Fernandez-Valverde S. L. Aguilera F. Ramos-Díaz R. A. (2018). Inference of Developmental Gene Regulatory Networks Beyond Classical Model Systems: New Approaches in the Post-Genomic Era. Integr. Comp. Biol. 58, 640–653. 10.1093/icb/icy061 29917089

[B18] Gao C. Zhang M. Chen L. (2020). The Comparison of Two Single-Cell Sequencing Platforms: BD Rhapsody and 10x Genomics Chromium. Curr. Genomics 21, 602–609. 10.2174/1389202921999200625220812 33414681 PMC7770630

[B19] Gilbert S. F. Barresi M. J. F. (2017). Developmental Biology, 11th Edition 2016. Am. J. Med. Genet. 173, 1430. 10.1002/ajmg.a.38166

[B20] Glass C. K. Rosenfeld M. G. (2000). The Coregulator Exchange in Transcriptional Functions of Nuclear Receptors. Available at: www.genesdev.org . 10652267

[B21] Haghverdi L. Büttner M. Wolf F. A. Buettner F. Theis F. J. (2016). Diffusion Pseudotime Robustly Reconstructs Lineage Branching. Nat. Methods 13, 845–848. 10.1038/nmeth.3971 27571553

[B22] Han X. Wang R. Zhou Y. Fei L. Sun H. Lai S. (2018). Mapping the Mouse Cell Atlas by Microwell-Seq. Cell 172, 1091–1107. e17. 10.1016/j.cell.2018.02.001 29474909

[B23] Hao Y. Hao S. Andersen-Nissen E. Mauck W. M. Zheng S. Butler A. (2020). Integrated Analysis of Multimodal Single-Cell Data Cell 24;184 (13), 3573–3587.e29. 10.1016/j.cell.2021.04.048 PMC8238499 34062119

[B24] Hayashi T. Ozaki H. Sasagawa Y. Umeda M. Danno H. Nikaido I. (2018). Single-cell Full-Length Total RNA Sequencing Uncovers Dynamics of Recursive Splicing and Enhancer RNAs. Nat. Commun. 9, 619. 10.1038/s41467-018-02866-0 29434199 PMC5809388

[B25] Huynh-Thu V. A. Geurts P. (2018). dynGENIE3: Dynamical GENIE3 for the Inference of Gene Networks from Time Series Expression Data. Sci. Rep. 8, 3384. 10.1038/s41598-018-21715-0 29467401 PMC5821733

[B26] Iglesias-Martinez L. F. Kolch W. Santra T. (2016). BGRMI: A Method for Inferring Gene Regulatory Networks from Time-Course Gene Expression Data and its Application in Breast Cancer Research. Sci. Rep. 6, 37140. 10.1038/srep37140 27876826 PMC5120305

[B27] Jin S. Guerrero-Juarez C. F. Zhang L. Chang I. Ramos R. Kuan C. H. (2021). Inference and Analysis of Cell-Cell Communication Using CellChat. Nat. Commun. 12, 1–20. 10.1038/s41467-021-21246-9 33597522 PMC7889871

[B28] Jolma A. Yan J. Whitington T. Toivonen J. Nitta K. R. Rastas P. (2013). DNA-Binding Specificities of Human Transcription Factors. Cell 152, 327–339. 10.1016/j.cell.2012.12.009 23332764

[B29] Kamitani M. Kashima M. Tezuka A. Nagano A. J. (2019). Lasy-Seq: A High-Throughput Library Preparation Method for RNA-Seq and its Application in the Analysis of Plant Responses to Fluctuating Temperatures. Sci. Rep. 9, 7091. 10.1038/s41598-019-43600-0 31068632 PMC6506593

[B30] Kashima M. Kamitani M. Nomura Y. Hirata H. Nagano A. J. (2020). DeLTa-Seq: Direct-Lysate Targeted RNA-Seq from Crude Tissue Lysate. (8/15 Words) Running Title: Development of Direct-Lysate Targeted RNA-Seq Method Corresponding Author. bioRxiv. 10.1101/2020.09.15.299180

[B31] Kilfoil M. L. Lasko P. Abouheif E. (2009). Stochastic Variation: From Single Cells to Superorganisms. HFSP J. 3, 379–385. 10.2976/1.3223356 20514130 PMC2839810

[B32] Klein A. M. Mazutis L. Akartuna I. Tallapragada N. Veres A. Li V. (2015). Droplet Barcoding for Single-Cell Transcriptomics Applied to Embryonic Stem Cells. Cell 161, 1187–1201. 10.1016/j.cell.2015.04.044 26000487 PMC4441768

[B33] Ko D. K. Brandizzi F. (2020). Network-Based Approaches for Understanding Gene Regulation and Function in Plants Plant J. 104 (2), 302–317. 10.1111/tpj.14940 32717108 PMC8922287

[B34] Krouk G. Mirowski P. LeCun Y. Shasha D. E. Coruzzi G. M. (2010). Predictive Network Modeling of the High-Resolution Dynamic Plant Transcriptome in Response to Nitrate. Genome Biol. 11, R123. 10.1186/gb-2010-11-12-r123 21182762 PMC3046483

[B35] Kumar M. P. Du J. Lagoudas G. Jiao Y. Sawyer A. Drummond D. C. (2018). Analysis of Single-Cell RNA-Seq Identifies Cell-Cell Communication Associated with Tumor Characteristics. Cell Rep 25, 1458e4–1468. 10.1016/j.celrep.2018.10.047 30404002 PMC7009724

[B36] Lam K. Y. Westrick Z. M. Müller C. L. Christiaen L. Bonneau R. (2016). Fused Regression for Multi-Source Gene Regulatory Network Inference. Plos Comput. Biol. 12, e1005157–23. 10.1371/journal.pcbi.1005157 27923054 PMC5140053

[B37] Li H. Durbin R. (2009). Fast and Accurate Short Read Alignment with Burrows-Wheeler Transform. Bioinformatics 25, 1754–1760. 10.1093/bioinformatics/btp324 19451168 PMC2705234

[B38] Li Y. Yang H. Zhang H. Liu Y. Shang H. Zhao H. (2020). Decode-Seq: A Practical Approach to Improve Differential Gene Expression Analysis. Genome Biol. 21, 66. 10.1186/s13059-020-01966-9 32200760 PMC7087377

[B39] Mathelier A. Zhao X. Zhang A. W. Parcy F. Worsley-Hunt R. Arenillas D. J. (2014). JASPAR 2014: An Extensively Expanded and Updated Open-Access Database of Transcription Factor Binding Profiles. Nucleic Acids Res. 42, D142–D147. 10.1093/nar/gkt997 24194598 PMC3965086

[B40] Matsumoto H. Kiryu H. Furusawa C. Ko M. S. H. Ko S. B. H. Gouda N. (2017). SCODE: An Efficient Regulatory Network Inference Algorithm from Single-Cell RNA-Seq during Differentiation. Bioinformatics 33, 2314–2321. 10.1093/bioinformatics/btx194 28379368 PMC5860123

[B41] Matys V. Kel-Margoulis O. V. Fricke E. Liebich I. Land S. Barre-Dirrie A. (2006). TRANSFAC and its Module TRANSCompel: Transcriptional Gene Regulation in Eukaryotes. Nucleic Acids Res. 34, D108–D110. 10.1093/nar/gkj143 16381825 PMC1347505

[B42] Muir P. Li S. Lou S. Wang D. Spakowicz D. J. Salichos L. (2016). Erratum to: The Real Cost of Sequencing: Scaling Computation to Keep Pace with Data Generation. Genome Biol. 17, 78. 10.1186/s13059-016-0961-9 27125642 PMC4850727

[B43] Ogami K. Yamaguchi R. Imoto S. Tamada Y. Araki H. Print C. (2012). Computational Gene Network Analysis Reveals TNF-Induced Angiogenesis. BMC Syst. Biol. 6 (Suppl. 2), S12–S18. 10.1186/1752-0509-6-S2-S12 PMC3521175 23281897

[B44] Patro R. Duggal G. Love M. I. Irizarry R. A. Kingsford C. (2017). Salmon Provides Fast and Bias-Aware Quantification of Transcript Expression. Nat. Methods 14, 417–419. 10.1038/nmeth.4197 28263959 PMC5600148

[B45] Percie du Sert N. Ahluwalia A. Alam S. Avey M. T. Baker M. Browne W. J. (2020). Reporting Animal Research: Explanation and Elaboration for the ARRIVE Guidelines 2.0. PLOS Biol. 18, e3000411. 10.1371/journal.pbio.3000411 32663221 PMC7360025

[B46] Pujato M. Kieken F. Skiles A. A. Tapinos N. Fiser A. (2014). Prediction of DNA Binding Motifs from 3D Models of Transcription Factors; Identifying TLX3 Regulated Genes. Nucleic Acids Res. 42, 13500–13512. 10.1093/nar/gku1228 25428367 PMC4267649

[B47] R Core Team. R (2015). A Language and Environment for Statistical Computing. Vienna, Austria: R Foundation for Statis-tical Computing. Available at: https://www.r-project.org/ .

[B48] Sasagawa Y. Danno H. Takada H. Ebisawa M. Tanaka K. Hayashi T. (2018). Quartz-Seq2: A High-Throughput Single-Cell RNA-Sequencing Method that Effectively Uses Limited Sequence Reads. Genome Biol. 19, 29. 10.1186/s13059-018-1407-3 29523163 PMC5845169

[B49] Sasagawa Y. Nikaido I. Hayashi T. Danno H. Uno K. D. Imai T. (2013). Quartz-Seq: A Highly Reproducible and Sensitive Single-Cell RNA Sequencing Method, Reveals Non-Genetic Gene-Expression Heterogeneity. Genome Biol. 14, R31. 10.1186/gb-2013-14-4-r31 23594475 PMC4054835

[B50] Sebé-Pedrós A. Ariza-Cosano A. Weirauch M. T. Leininger S. Yang A. Torruella G. (2013). Early Evolution of the T-Box Transcription Factor Family. Proc. Natl. Acad. Sci. U S A. 110, 16050–16055. 10.1073/pnas.1309748110 24043797 PMC3791752

[B51] Sing T. Sander O. Beerenwinkel N. Lengauer T. (2005). ROCR: Visualizing Classifier Performance in R. Bioinformatics 21, 3940–3941. 10.1093/bioinformatics/bti623 16096348

[B52] Skoda A. M. Simovic D. Karin V. Kardum V. Vranic S. Serman L. (2018). The Role of the Hedgehog Signaling Pathway in Cancer: A Comprehensive Review./pmc/articles/PMC5826678/ 10.17305/bjbms.2018.2756 PMC5826678 29274272

[B53] Street K. Risso D. Fletcher R. B. Das D. Ngai J. Yosef N. (2018). Slingshot: Cell Lineage and Pseudotime Inference for Single-Cell Transcriptomics. BMC Genomics 19, 477. 10.1186/s12864-018-4772-0 29914354 PMC6007078

[B54] Stuart T. Butler A. Hoffman P. Hafemeister C. Papalexi E. Mauck W. M. (2019). Comprehensive Integration of Single-Cell Data. Cell 177, 1888–1902. 10.1016/j.cell.2019.05.031 31178118 PMC6687398

[B55] Treutlein B. Brownfield D. G. Wu A. R. Neff N. F. Mantalas G. L. Espinoza F. H. (2014). Reconstructing Lineage Hierarchies of the Distal Lung Epithelium Using Single-Cell RNA-Seq. Nature 509, 371–375. 10.1038/nature13173 24739965 PMC4145853

[B56] Tritschler S. Büttner M. Fischer D. S. Lange M. Bergen V. Lickert H. (2019). Concepts and Limitations for Learning Developmental Trajectories from Single Cell Genomics. Development 27;146 (12), dev170506. 10.1242/dev.170506 31249007

[B57] Ujibe K. Nishimura K. Kashima M. Hirata H. (2021). Direct-TRI: High-Throughput RNA-Extracting Method for All Stages of Zebrafish Development. Bio-Protocol 11 (17), e4136. 10.21769/BioProtoc.4136 34604443 PMC8443459

[B58] Wang S. Karikomi M. Maclean A. L. Nie Q. (2019). Cell Lineage and Communication Network Inference via Optimization for Single-Cell Transcriptomics. Nucleic Acids Res. 47, e66. 10.1093/nar/gkz204 30923815 PMC6582411

[B59] Wei G. H. Badis G. Berger M. F. Kivioja T. Palin K. Enge M. (2010). Genome-Wide Analysis of ETS-Family DNA-Binding *In Vitro* and *In Vivo* . EMBO J. 29, 2147–2160. 10.1038/emboj.2010.106 20517297 PMC2905244

[B60] Weirauch M. T. Cote A. Norel R. Annala M. Zhao Y. Riley T. R. (2013). Evaluation of Methods for Modeling Transcription Factor Sequence Specificity. Nat. Biotechnol. 31, 126–134. 10.1038/nbt.2486 23354101 PMC3687085

[B61] Weirauch M. T. Yang A. Albu M. Cote A. G. Montenegro-Montero A. Drewe P. (2014). Determination and Inference of Eukaryotic Transcription Factor Sequence Specificity. Cell 158, 1431–1443. 10.1016/j.cell.2014.08.009 25215497 PMC4163041

[B62] Yoshino K. Nishijima R. Kawakatsu T. (2020). Low-Cost RNA Extraction Method for Highly Scalable Transcriptome Studies. Breed. Sci. 70 (4), 481–486. 10.1270/jsbbs.19170 32968351 PMC7495201

[B63] Zhang J. Zhu W. Wang Q. Gu J. Huang L. F. Sun X. (2019). Differential Regulatory Network-Based Quantification and Prioritization of Key Genes Underlying Cancer Drug Resistance Based on Time-Course RNA-Seq Data. PLOS Comput. Biol. 15, e1007435. 10.1371/journal.pcbi.1007435 31682596 PMC6827891

