# Supplementary material for: Intracellular and Intercellular Gene Regulatory Network Inference From Time-Course Individual RNA-Seq
Source: Front Bioinform. 2021 Nov 11;1:777299. doi: 10.3389/fbinf.2021.777299 (PMC9580923; doi:10.3389/fbinf.2021.777299)
Supplement: Supplementary file 2 [file DataSheet1.docx]

Supplementary Material


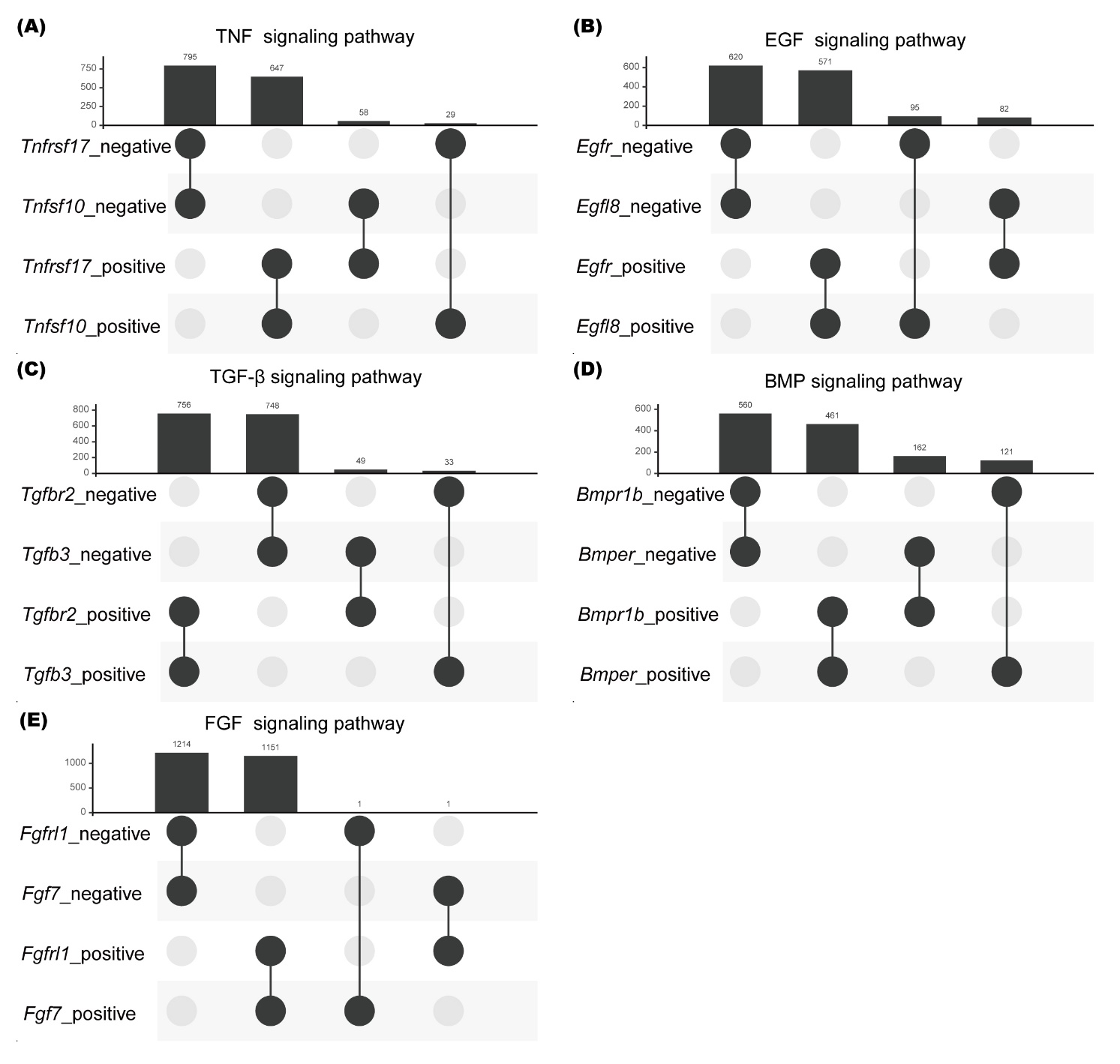


**Supplementary Figure 1. Overlaps of inferred downstream genes for ligand- and receptor-related genes.**

Upset plots of inferred downstream genes that were positively and negatively regulated by the representative ligand- and receptor-related genes. (A) TNF signaling pathway; (B) EGF signaling pathway; (C) TGFβ signaling pathway; (D) BMP signaling pathway; (E) FGF signaling pathway.

**
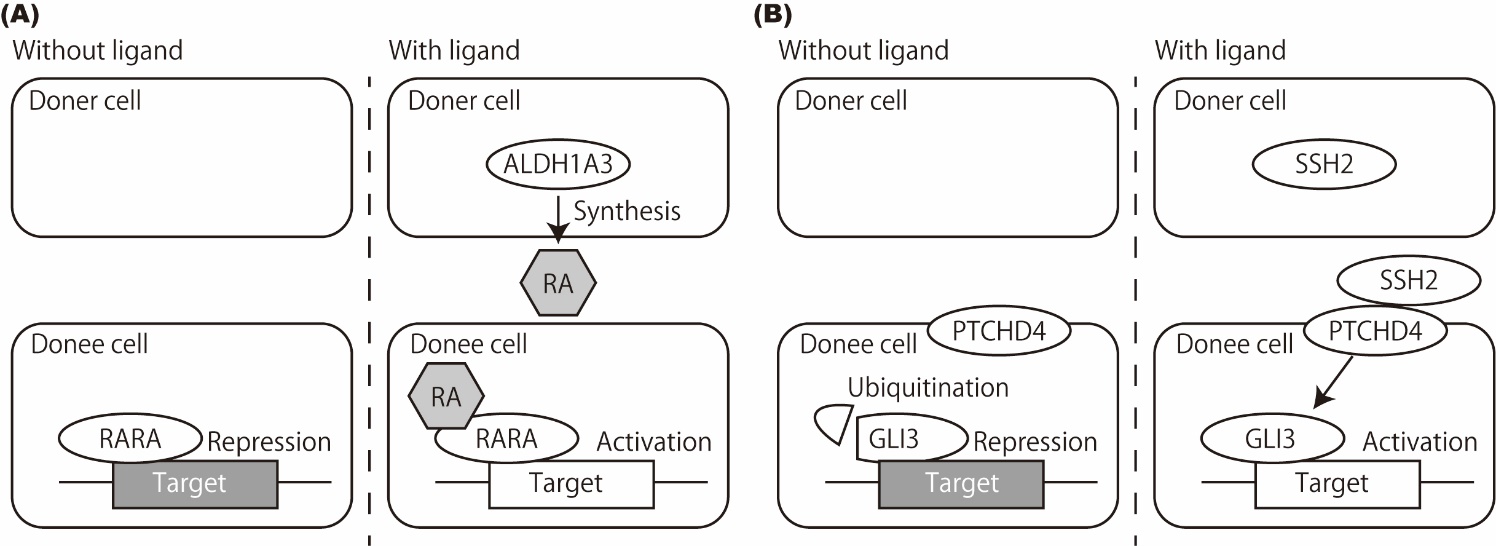
**

**Supplementary Figure 2. Schematic illustration of the retinoic acid and Hedgehog signaling pathway.**

(A) In the absence of retinoic acid (RA), the RA receptor (RARA) represses target genes. ALDH1A (ALDH1A3) synthesizes RA. In the presence of RA, RA receptor activates the target genes. (B) In the absence of Hedgehog ligand (SSH2), GLI protein (GLI3) is ubiquitinated to function as a repressor of the target genes. In the presence of Hedgehog ligand, PATCHED (PTCHD4) inhibits GLI ubiquitination to activate the target genes.

**
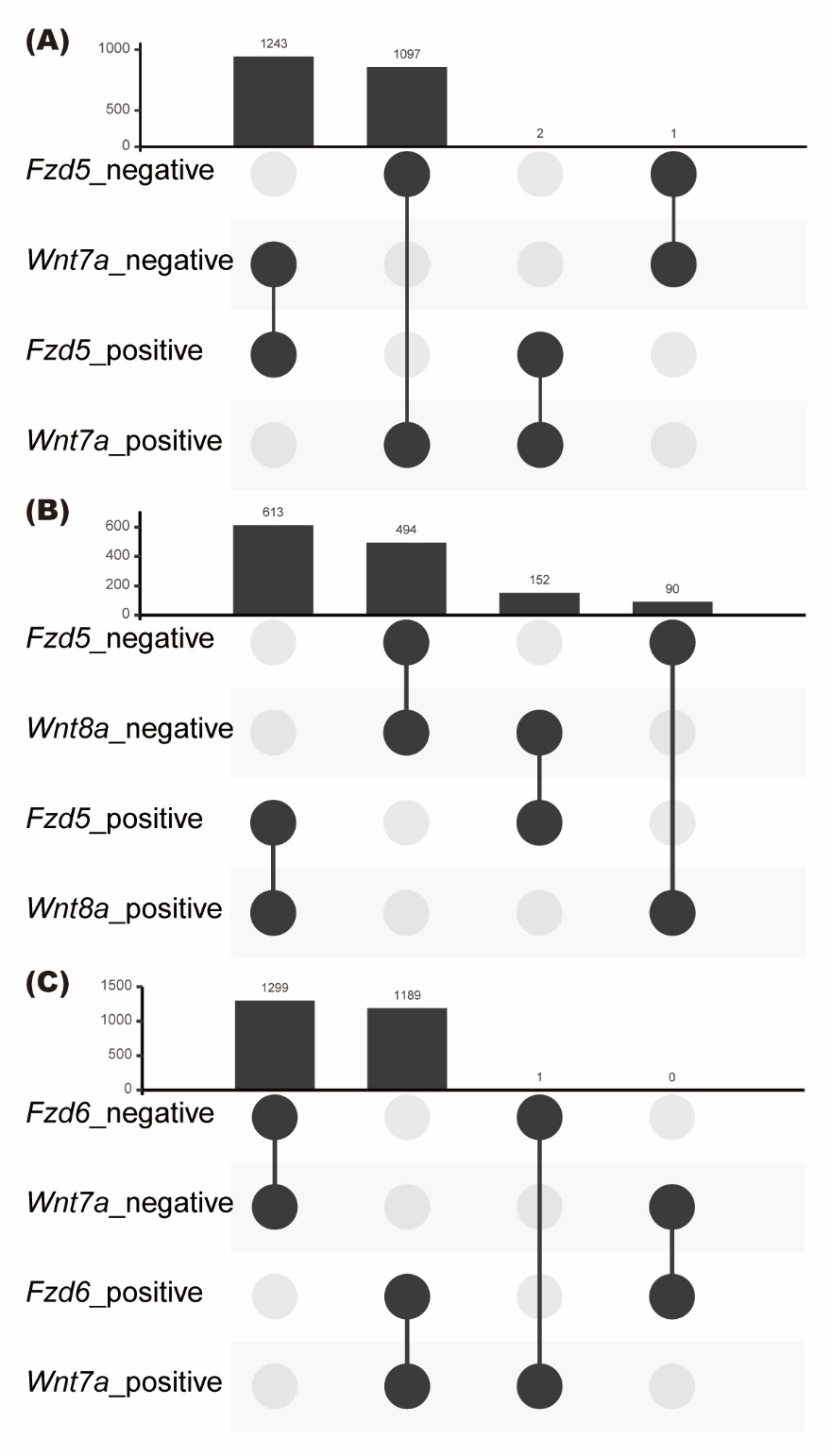
**

**Supplementary Figure 3. Overlaps of inferred downstream genes of *Wnt7a, Wnt8a, Fzd5,* and *Fzd6.***

Upset plots of inferred downstream genes that were positively and negatively regulated by the representative *Wnt7a* and *Fzd5* (A), *Wnt8a* and *Fzd5* (B), and *Wnt7a* and *Fzd6* (C).


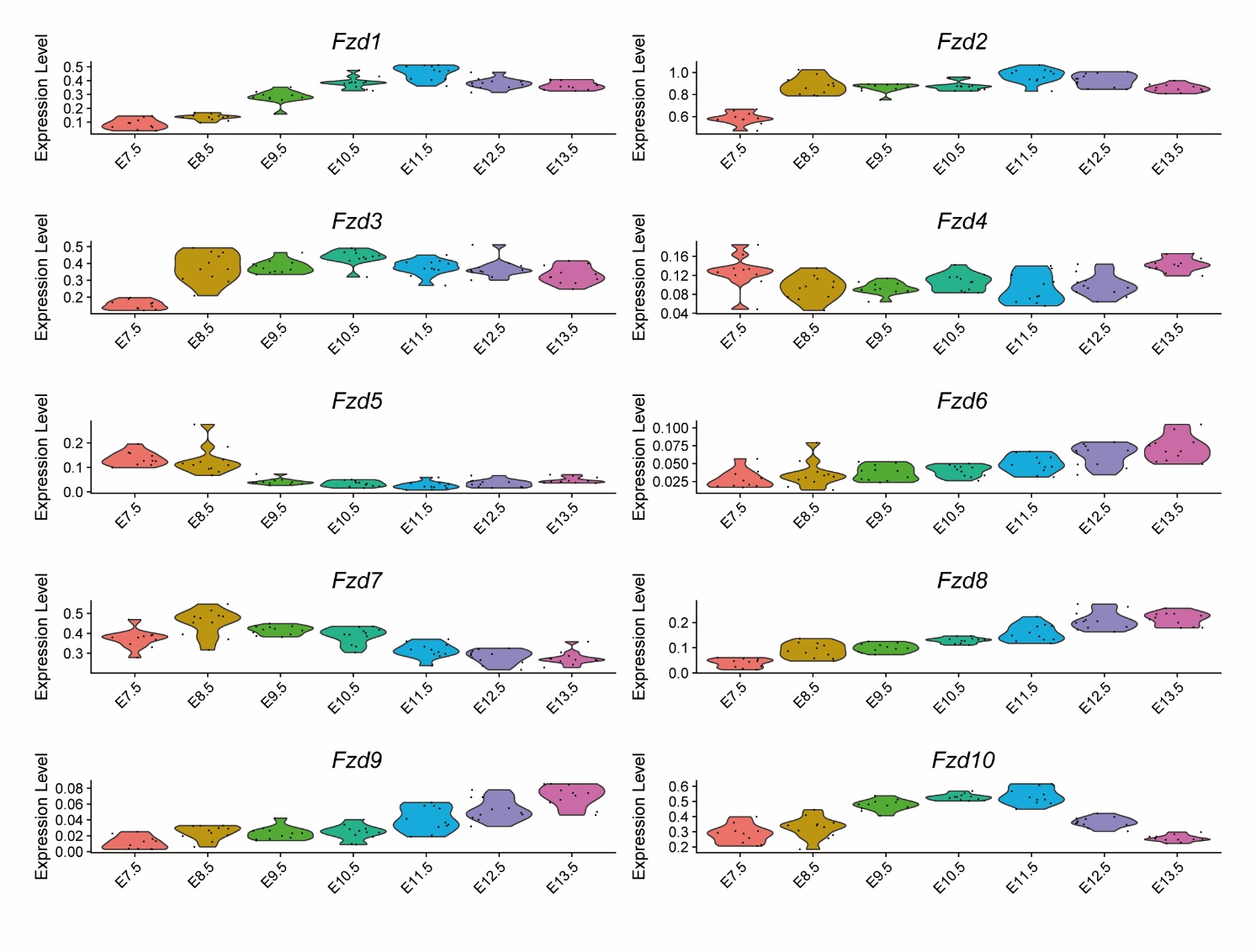
 **Supplementary Figure 4. Expression dynamics of the *Fzd* genes.**

Violin plots for the relative expression of the *Fzd* genes from E7.5 to E13.5.


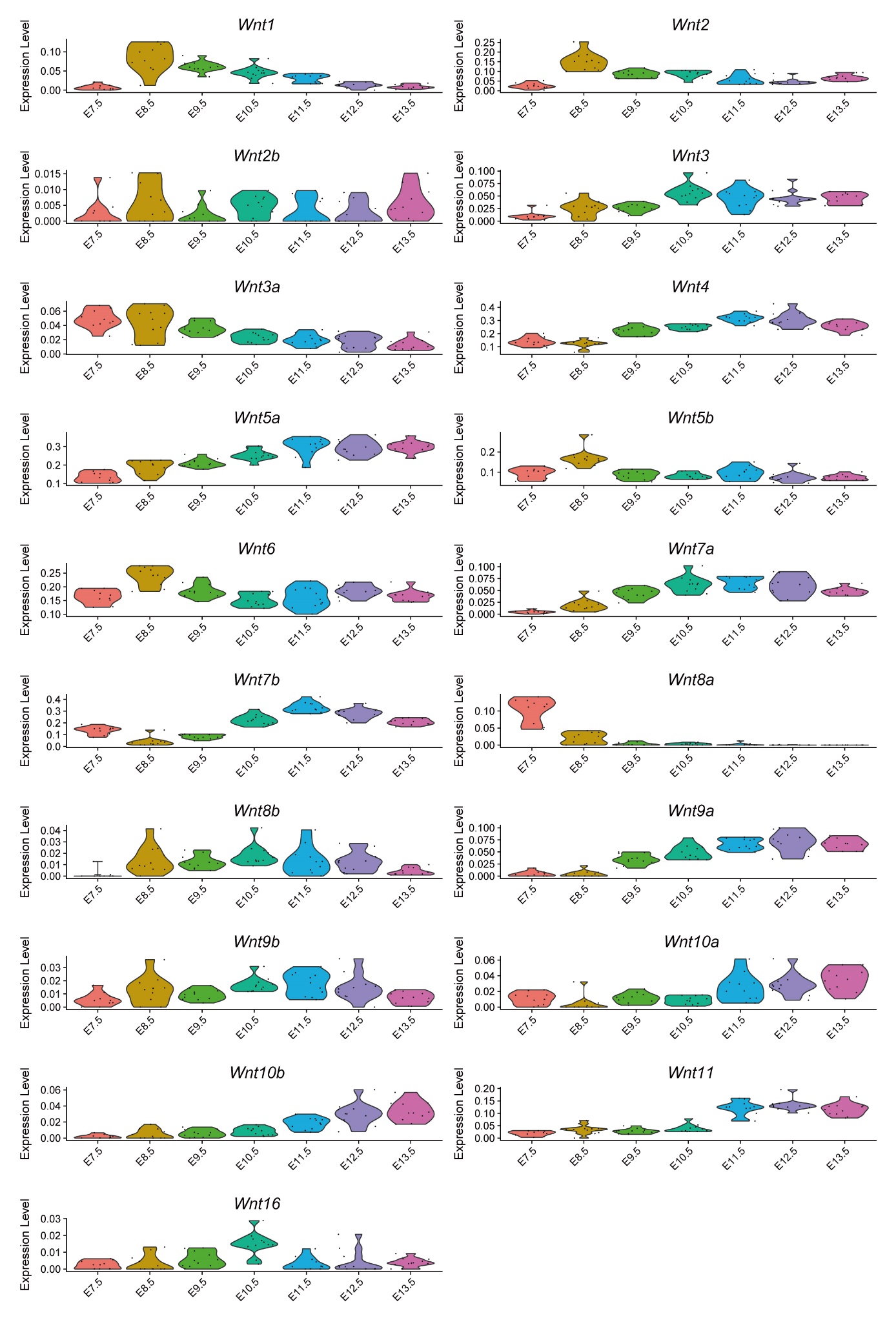


**Supplementary Figure 5. Gene expression dynamics of *Wnt* genes.**

Violin plots for the relative expression of *Wnt* genes from E7.5 to E13.5.


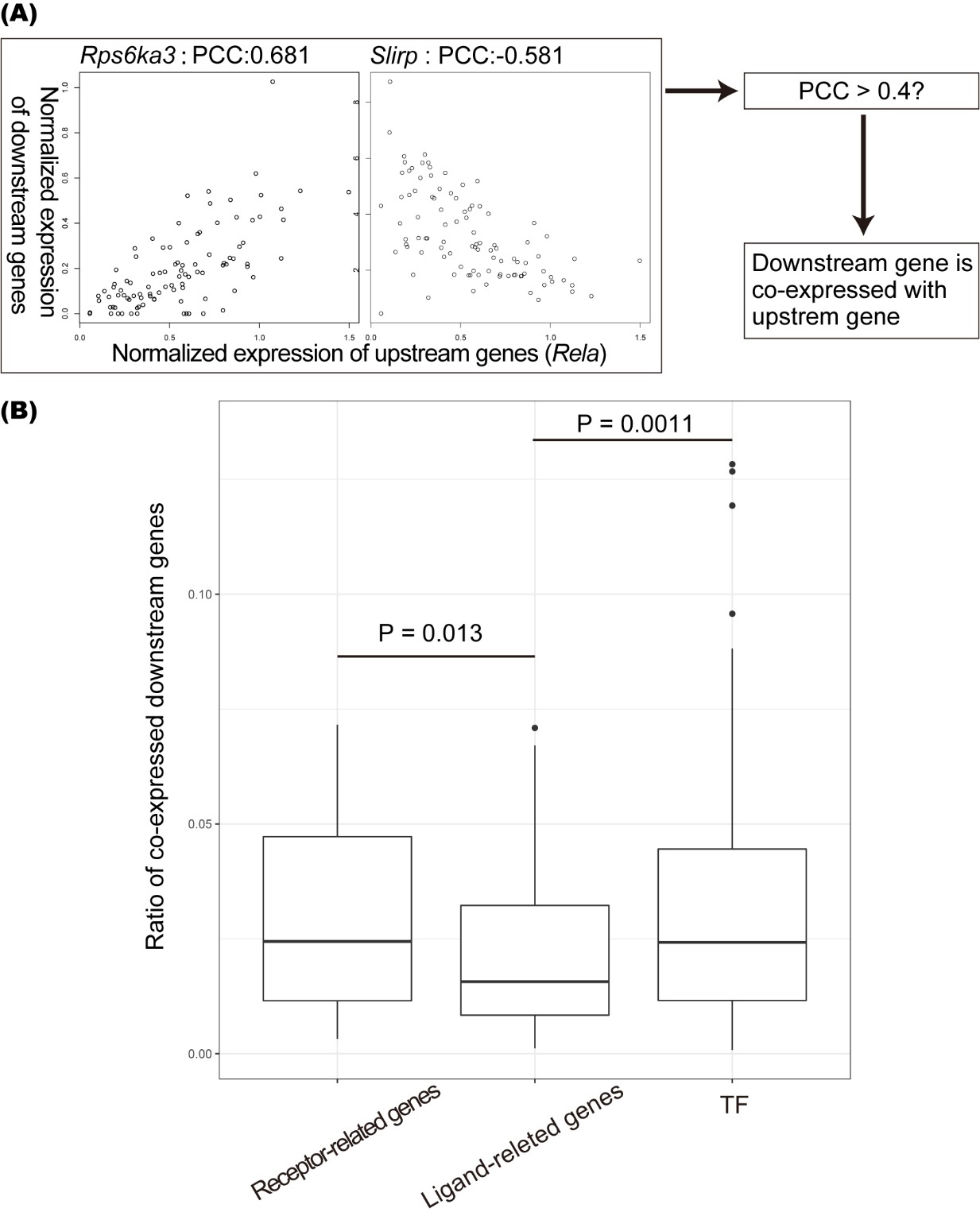


**Supplementary Figure 6. Ratio of co-expression of upstream and downstream genes.**

(A) Co-expression of upstream and downstream genes was assessed based on Pearson’s correlation coefficient (PCC) of normalized gene expression levels of upstream and downstream genes in a publicly available mouse cell atlas (Han et al., 2018). (B) Boxplot of ratio of co-expression of upstream and downstream genes. P-value was calculated using Student’s *t*-test.


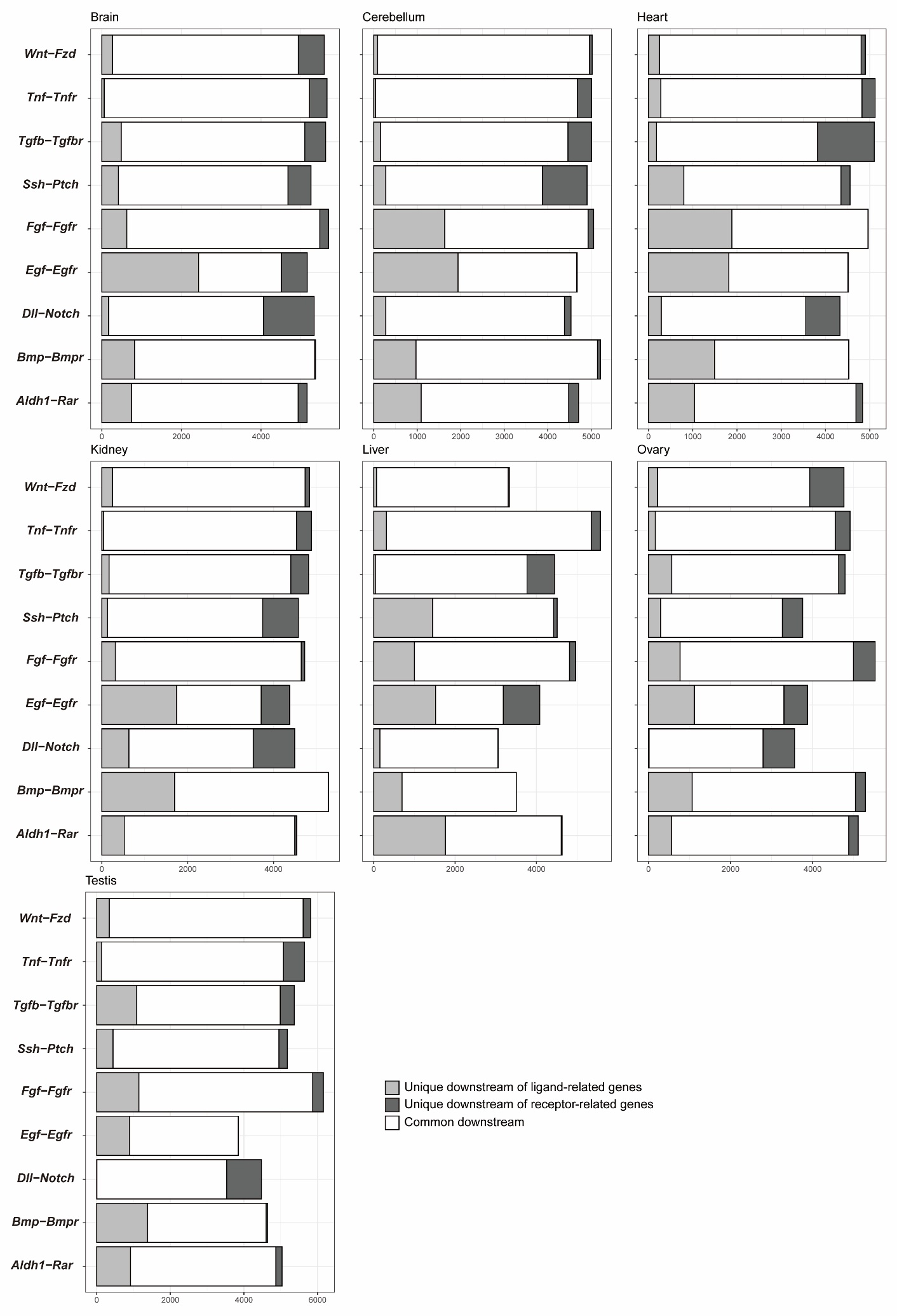


**Supplementary Figure 7. Overlap of inferred genes downstream of ligand- and receptor-related genes from a publicly available time-course organ-level individual RNA-Seq.**

Bar plot of the number of inferred downstream genes that are common and unique for each ligand–receptor pair from each mouse organ.
